# The effect of cadmium exposition on the structure and mechanical properties of rat incisors

**DOI:** 10.1371/journal.pone.0215370

**Published:** 2019-04-12

**Authors:** Izabela Świetlicka, Ewa Tomaszewska, Siemowit Muszyński, Jose Luis Valverde Piedra, Michał Świetlicki, Adam Prószyński, Krystian Cieślak, Dariusz Wiącek, Sylwia Szymańczyk, Daniel Kamiński

**Affiliations:** 1 Department of Biophysics, Faculty of Production Engineering, University of Life Sciences in Lublin, Lublin, Poland; 2 Department of Animal Physiology, Faculty of Veterinary Medicine, University of Life Sciences in Lublin, Lublin, Poland; 3 Department of Preclinical Veterinary Sciences, Faculty of Veterinary Medicine, University of Life Sciences in Lublin, Lublin, Poland; 4 Department of Applied Physics, Faculty of Mechanical Engineering, Lublin University of Technology, Lublin, Poland; 5 Institute of Renewable Energy Engineering, Faculty of Environmental Engineering, Lublin University of Technology, Lublin, Poland; 6 Department of Physical Properties of Plant Materials, Institute of Agrophysics, Polish Academy of Sciences, Lublin, Poland; 7 Department of Crystallography, Faculty of Chemistry, Maria Curie-Skłodowska University, Lublin, Poland; Max Planck Institute for Evolutionary Anthropology, GERMANY

## Abstract

Alterations in the structure and mechanical properties of teeth in adult Wistar rats exposed to cadmium were investigated. Analyses were conducted on two sets of incisors from female and male specimens, that were intoxicated with cadmium (n = 12) or belonged to the control (n = 12). The cadmium group was administered with CdCl_2_ dissolved in drinking water with a dose of 4mg/kg_bw_ for 10 weeks. The oral intake of cadmium by adult rats led to the range of structural changes in enamel morphology and its mechanical features. A significant increase of cadmium levels in the teeth in comparison to the control, a slight shift in the colour and reduction of pigmented enamel length, higher surface irregularity, a decrease of hydroxyapatite crystals size in the c-axis and simultaneous increase in pigmented enamel hardness were observed. The extent of these changes was sex-dependent and was more pronounced in males.

## Introduction

Enamel, the hardest material in the body consists on average of 95% hydroxyapatite (HA) crystals (Ca_10_(PO_4_)_6_(OH)_2_), 4% water and 1% organic matter [1]. The organic phase is composed of proteins (amelogenins, ameloblastin, enamelin, and tuftelin) and minor concentrations of proteoglycans and lipids [2–4]. Enamel is characterised by strict hierarchical organization. Hydroxyapatite (HA) crystals, tightly packed into groups and bound together, form prisms, that along with the protein-rich interprism matter are assembled into prism bands [1, 5]. Enamel formation process (amelogenesis) is highly ordered and runs through three functional phases: pre-secretory, secretory and maturation stage [2, 3]. At each stage amelogenesis may be disturbed by environmental or developmental factors and each disturbance may translate into changes in the final structure of the tissue.

The numerous determinants which could influence amelogenesis, include the environmental factors. They include such parameters as temperature, pH, radiation, and, finally, the presence of trace elements. While some trace elements are essential for life and play a key role in metabolic processes, others can contribute to the serious tissue damage, disturbing their functions and triggering a wide range of diseases [6, 7].

Cadmium metal (Cd) exists in its natural form usually with zinc and lead in sulphide ores and is widely used in the industry, e.g. in the production processes of alloys or highly specialized electronic products. Frequently, due to weathering or human activities, potentially harmful compounds of cadmium are released into the environment [8] and can be introduced into living organisms through oral path (water and intake of contaminated food) and/or inhalation [9]. Cadmium shows mutagenic, genotoxic and teratogenic effects in humans and animals [10]. Its negative actions also include developmental disorders of the skeleton [11–14]. Cadmium affects bone tissue cells [15, 16], influencing the bone remodelling process, i.a. by inducing activation of caspase-3 and -8, that are involved in osteoblast apoptosis [17]. Additionally, due to Cd intake by cells in the kidney tubules and its accumulation in the mitochondria the process of vitamin D metabolism is impaired and, as a consequence, the absorption of calcium from the intestinal tract is reduced [18].

Although teeth are not the primary target organs for cadmium, unlike kidneys and liver, it is accumulated in their hard tissues [19] influencing their development [20] and morphology [21–23]. Cadmium is able to substitute Ca^2+^ ions in HA crystals [24] and calcium proteins [25]. Its action includes decreasing c-axis spacing, causing crystal imperfections and disturbing its symmetry as well as altering proteolysis of enamel matrix proteins, as observed both in *in vitro* [21, 26, 27] and in *in vivo* model studies [21, 26, 28]. The substitution can occur because both metals are divalent cations, have the same overall charge and very close ionic radii [27].

Since Cd actions on enamel might be considered as possible inductors of changes in the structural properties of teeth, the aim of the presented research was to assess alterations in the structure and mechanical properties of incisors of adult Wistar rats exposed to cadmium action over a defined period of time. Changes in enamel morphology were studied using chroma measurements, atomic force microscopy (AFM) and scanning electron microscopy (SEM) imaging. The crystalline structure was assessed with the application of X-ray diffraction (XRD) whereas the mechanical properties of teeth were checked with a three-point bending test and micro hardness measurements. The mineral content was estimated with inductively coupled plasma optical emission spectrometry (ICP-OES). Since the action of cadmium on living organisms is proved to be related to sex in many aspects [29–31] male and female rats were investigated separately to assess possible sex-dependent changes. The analyses were conducted on rat incisors because it has been proved that cadmium intoxication alters their pigmentation and induces enamel hypoplasia discernible as surface cross-striation [32, 33], which makes them a suitable model in Cd toxicity studies.

## Materials and methods

The experiment was approved by the Bioethics Commission at the University of Life Sciences in Lublin, Poland (reference number 83/2015) and was conducted in accordance with EU Directive 2010/63/EU for animal experiments. The animals were purchased from the certified Animal Husbandry in Brwinów, Warsaw, Poland.

**Fig 1:**
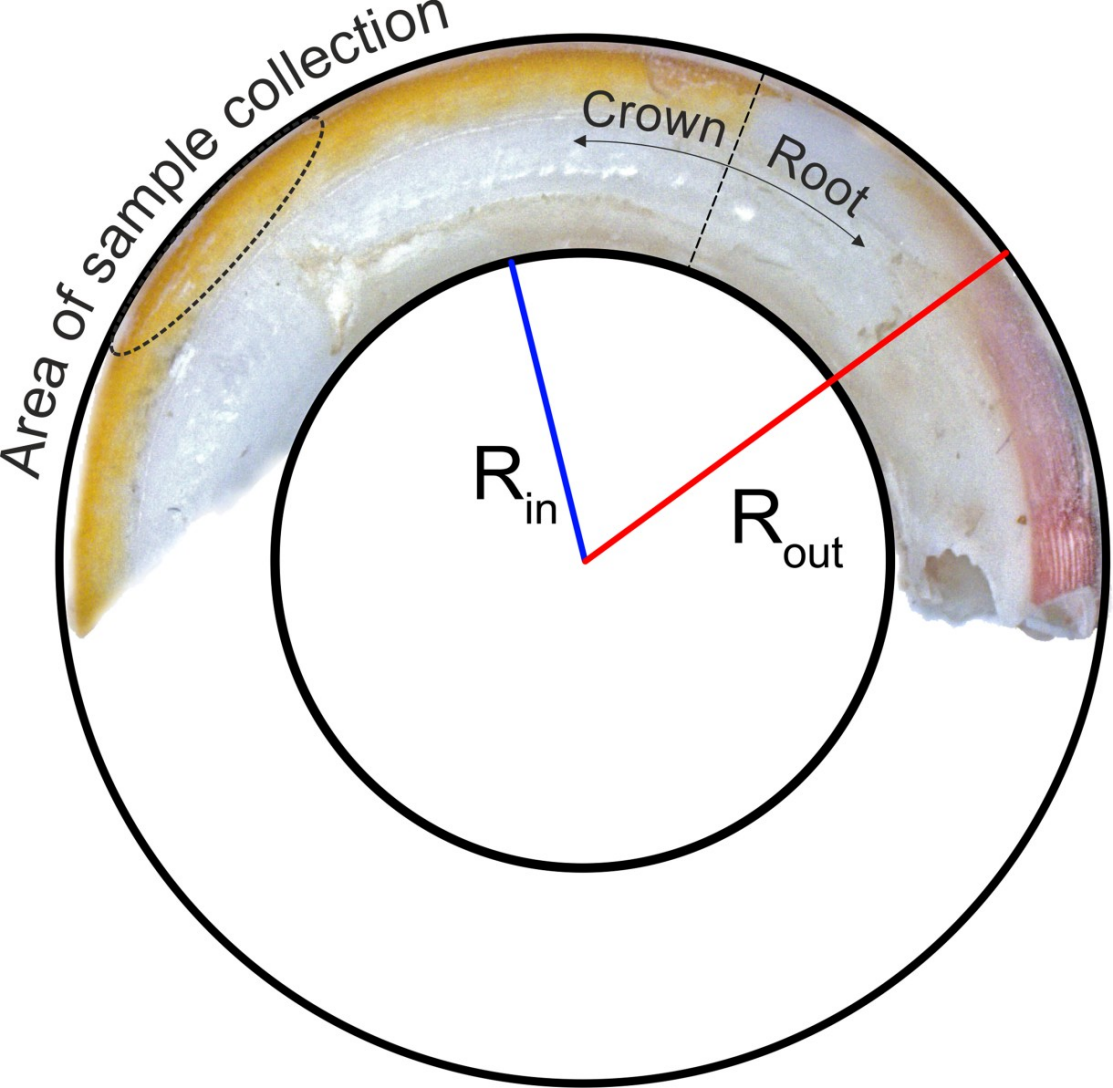
Incisor with marked crown and root, area of sample collection and approximation of incisor curvature by homocentric circles with outer R_out_ and inner R_in_ radiuses.

The research material included two sets of upper incisors from adult female and male albino Wistar rats which belonged to the control or were exposed to cadmium over a period of 10 weeks. Each group consisted of 24 teeth (Σ = 48): 12 from female (f) and 12 from male (m) specimens. After preparation and initial measurements (mass, length, thickness), the teeth from each group were randomly divided into two equal sets. One set of teeth (n = 24) was subjected to chroma measurements, AFM and hardness assessment and next mineralised and used for mineral content determination with ICP-OES application, while the second set (n = 24) was subjected to the three-point bending test, XRD analysis, and finally SEM imaging. Chroma, AFM, micro hardness, XRD and SEM measurements were conducted on the labial surface or its parts covered by pigmented enamel, approximately at one-third of the tooth length measured from the tip (Fig 1). Three-point bending test and mineral content analysis were performed on the whole tooth. The detailed description of experimental procedures is provided below.

### Animals

The studies were conducted on 24 clinically healthy adult male (n = 12) and female (n = 12) albino Wistar rats weighing 230 g and 330 g (± 20 g) respectively and being in the age of 3 months at the beginning of the experiment. The female and male rats were kept in separate standard cages and were allowed to acclimate for 14 days. The rats were kept in a well-ventilated room at an ambient temperature of 24.0°C (± 2.0°C) and relative humidity of 50% (± 10%), under 12 h light/dark cycle, supplied with feed and water *ad libitum*. Two groups of rats were formed: the control group (c, n = 12, 6 males and 6 females) fed a standard laboratory rodents diet, formulated to meet the minimal nutritional requirements specified in AIN-93M [34] and filtered water, and the cadmium group (Cd, n = 12, 6 males and 6 females) fed with the same diet and exposed to cadmium in the form of CdCl_2_ (Sigma-Aldrich St. Louis, MO, USA) solution given via drinking water. The CdCl_2_ solution was prepared in filtered water at the concentration of 19.4 mmol/l, in order to reach the dose of 4 mg/kg_bw_. Before the beginning of the experiment, the total water consumption during 24 hours was determined. The volume of water left in the bottles was measured to follow cadmium consumption and, along with body weight (measured twice a week), used to maintain the proper amount of cadmium given in drinking water [35]. The drinking water and the solutions of CdCl_2_ were replaced twice a week with freshly prepared ones which allows to maintain the Cd concentration at near to the study value. At the end of the experiment, the animals were fasted for approximately 12 h and euthanized by CO_2_ overdose. Since the rate of upper incisors growth hovers on average at approximately 2.2 mm per week and it takes about 40–50 days for new tooth tissue formed at the base to reach the tip [36, 37], a 10-week period guarantees that the teeth of the rats from the Cd group grew in the presence of cadmium and all the analyses were conducted on cadmium-intoxicated teeth.

### Teeth sample preparation and initial measurements

The incisor teeth were extracted without visible fractures. After extraction, teeth were disinfected in aqueous 0.5% Chloramine-T solution (Poch S.A., Gliwice, Poland) for 24 h and stored in a 0.9% NaCl solution at 4°C for no longer than 7 days [38–40]. Before the examination, each tooth was immersed for 5 min in deionized water and then dried in a desiccator for 24 h under vacuum conditions to remove water from the tooth’s exterior surface. All the reagents used were of analytical grade and were dissolved in deionized water (16 MΩ) purified via a Mili-Q purification system (Milipore, Bedford, MA, USA).

The teeth were weighed and measured before an examination. The shape of the upper incisors and the length of incisors’ clinical crowns (incisal edge) along with the pigmented enamel length were evaluated using the digital microscope (VHS-2000, Keyence Corp., Osaka, Japan). The curvature of the incisor was approximated by the homocentric circles with outer (R_out_) and inner (R_in_) radiuses as presented in Fig 1. The thickness of the teeth was calculated as a difference between both radiuses.

### Chroma measurements

Minolta CR-221 Chroma Meter (Minolta, Osaka, Japan) with 45° circumferential illumination and 0° viewing angle geometry measurement, aperture size Ø 3 mm and D65 light source (pulsed xenon lamp) was used to assess enamel colour both for the control and the cadmium groups. Before analysis, the instrument was calibrated with a white ceramic tile (CIE L* = 99.25; a* = –0.60; b* = 1.87) according to the manufacturer's recommendations. The measurements were performed in a darkened room to exclude external light sources. The total number of examined teeth was n = 24 (n = 6 for each subgroup) and colour coordinates were recorded three times for each tooth to ensure accurate measurement repeatability, and the mean L*, a* and b* values, indicating respectively lightness, which ranges from 0 (black) to 100 (white) and colour directions from red (+a*) to green (-a*) and from yellow (+b*) to blue (-b*) [41, 42], were determined. On the basis of the received parameters, C (chroma) and h (hue) values (CIE LCh) were calculated from Eqs 1 and 2 to provide a better colour indication.

$$C=\sqrt{a^{*2}+b^{*2}},$$ (1)

$$h={tg}^{-1}\left(\frac{b^{*}}{a^{*}}\right),$$ (2)

Additionally, the distance ΔE was determined according to Eq 3 and, in accordance with the National Bureau of Standards (NBS) recommendation, multiplied by 0.92 to obtain NBS values [43, 44].

$$\Delta E=\sqrt{{\left(L_{1}^{*}-L_{2}^{*}\right)}^{2}+{\left(a_{1}^{*}-a_{2}^{*}\right)}^{2}+{\left(b_{1}^{*}-b_{2}^{*}\right)}^{2}}$$ (3)

ΔE informs if it is possible to distinguish between two colours i.e. displays the difference as a single value for colour and lightness, whereas NBS values approximate the human perception system, according to which changes in colours can be observed on the level from extremely marked change (6–12), by marked change (3–6) and perceivable change (1.5–3) to a slight change (0.5–1.5).

### Atomic force microscopy imaging and roughness assessment

Atomic force microscopy measurements were taken on the labial surface covered by pigmented enamel. The roots were cut off and the remaining crowns were mounted on the plates by an adhesive tape. The surface was first examined under an optical microscope in order to determine 5 regions, which were next submitted to AFM imaging. The measurements were conducted in the tapping mode at room temperature, under atmospheric conditions at a relative humidity of 25% by Multimode 8 atomic force microscope (Bruker, Billerica, MA, USA). For each tooth five areas in the size of 10 μm×10 μm were investigated with a slow scan rate of 1 Hz and with a resolution of 512×512 pixels per image. Each scan was done along the long axis of the tooth. The enamel surface was pictured in height, error and phase domains. The parameters describing surface roughness (average roughness S_a_ and root mean square roughness S_q_) were determined for a height function Z_ij_(x,y) defined over a certain XY plane, according to the Eqs 4 and 5:

$$S_{a}=\frac{1}{N_{x}N_{y}}\sum_{j=1}^{N_{y}}\sum_{i=1}^{N_{x}}\left|Z_{ij}\right|,$$ (4)

$$S_{q}=\sqrt{\frac{1}{N_{x}N_{y}}\sum_{j=1}^{N_{y}}\sum_{i=1}^{N_{x}}Z_{ij}^{2}},$$ (5)

where N_x_ and N_y_ are the sampling rates along X and Y axis, respectively [45, 46].

### Scanning electron microscopy

To perform the morphological analysis of the surface of the teeth, scanning electron microscope (SU3500, Hitachi Ltd., Tokyo, Japan) operating at 20 kV in high vacuum conditions was used. Before introducing the samples to the SEM chamber, the teeth from the set previously examined in bending tests and XRD were cut, and small pieces of teeth with the surface covered by pigmented enamel were attached with a carbon tape to an aluminium stage. The samples were coated with an ultra-thin gold (Au) film with ion sputtering equipment (Sputter Coater 108auto, Cressington Sci. Instr., Watford, UK). The enamel surface was imaged with x10 000 magnification.

### Micro hardness

Indentations were made on 24 pigmented enamel samples, using Anton Paar MHT-10 microindenter (Anton Paar, Graz, Austria) mounted to a metallurgical microscope (Axiotech, Carl Zeiss, Oberkochen, Germany) equipped with a CDD camera. The measurements were performed with Vickers indenter heads. Loads ranging from 0.01 to 4 N were used for the indentation time of 10 s. The offset of the diagonal tip was <0.25 μm and the load resolution was equal to 0.001 N. To avoid overlapping of surface stresses developed around the neighboring indentations, the separation between indentation diagonals was kept in the range of more than ten times the diagonal length of the indentation impressions. The indentation tests were conducted at a controlled humidity below 40% and temperature of 22 ^o^C. All the indentations were made on the prisms heads, 5 times for each sample. Photographs of indented samples with indentation imprints were recorded on a computer. The dimensions of indentation diagonals d, made at a particular load P were measured manually from the photographs, and the diagonal geometric mean d was calculated. The value of micro hardness H [Pa] was calculated from the P(d) data using the standard relation (Eq 6):

H=kPd2 (6)

where k is a geometrical conversion factor for the indenter (k = 1.854).

### Fracture force

The ultimate fracture force of the teeth was determined on the basis of the three-point bending test performed on the universal testing machine (Zwick/Roell 005, Zwick-Roell GmbH & Co. KG, Ulm, Germany). The incisors from the second set (n = 24 in total, 6 from each subgroup) were used. The examined tooth was placed in the frontal plane and the distance between supports was set at 3.0 mm. A load was applied at the incisal edge and at a constant rate of 5 mm/min until fracture.

### X-ray diffraction measurements

**Fig 2:**
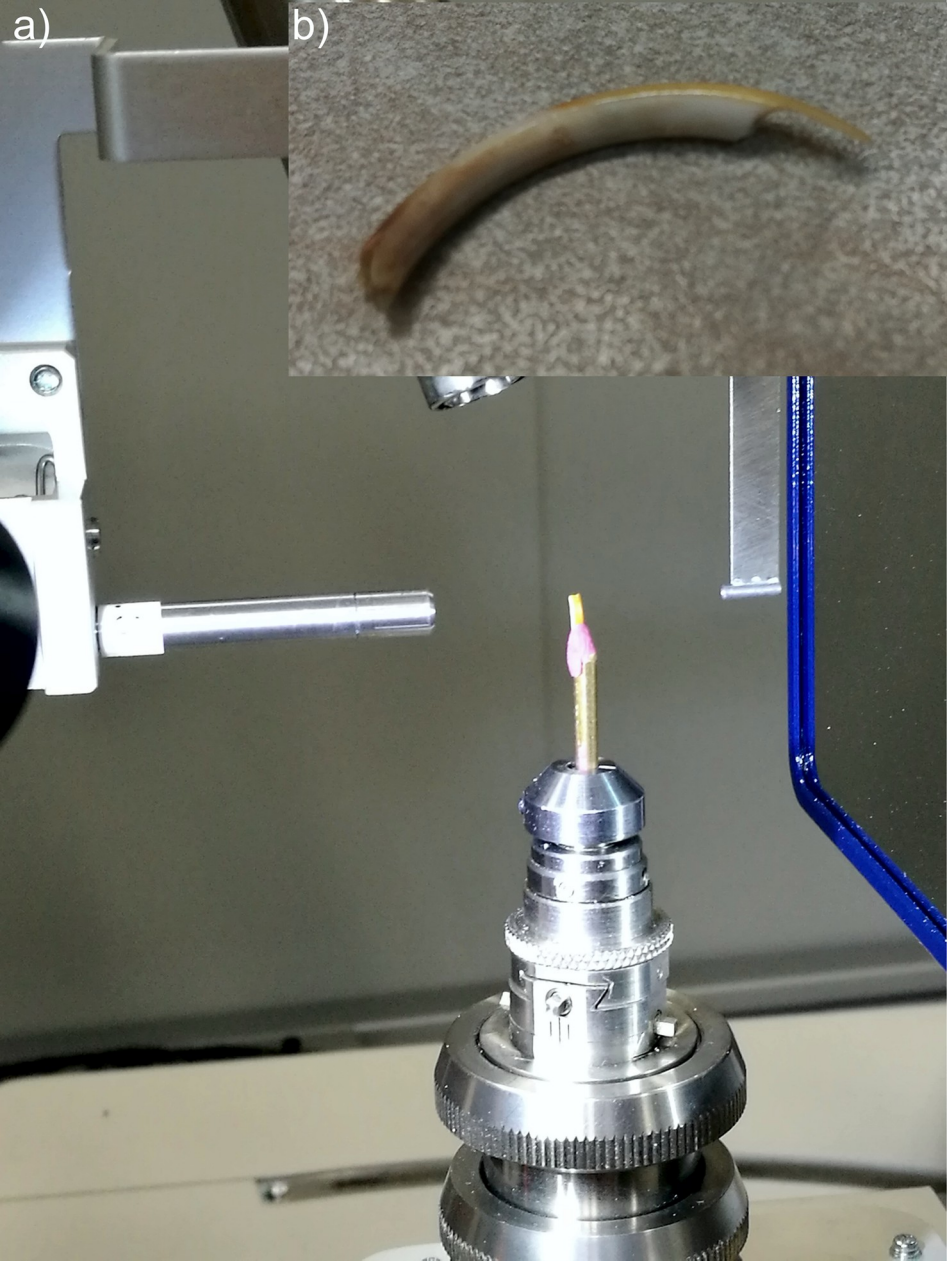
Incisor preparation for X-ray diffraction (a) and XRD measurements (b).

Prior to the examination, teeth were cut and polished until pigmented enamel surface became needle shaped as presented in Fig 2B. The samples were measured in θ-2θ geometry with Rigaku XtaLAB diffractometer (Fig 2A) equipped with MicroMax-007 HF rotated anode X-ray source and Pilatus 300 K area detector over a range of 6–110 ^o^ with a resolution of 0.078 ^o^ and 10 min counting time per frame.

The X-ray beam diameter was 0.1 mm. During measurement the sample was rotated around the direction perpendicular to the θ-2θ plane (around the z direction) for signal averaging from 0.1 mm sample slices. The measured frames were processed in CrysAlisPRO software. The mean size of the nanocrystallites was calculated according to the Scherrer equation (Eq 7) [47]

$$D=\frac{k\lambda }{\beta cos\theta },$$ (7)

where D is the mean size of the ordered crystalline domains, k is a constant related to the crystallite shape (0.9), β is the full width of the peak at half of the maximum intensity (FWHM) counting the apparatus broadening of 0.08 deg (limited by the detector resolution at 40 mm from the sample) and λ is the wavelength of X-ray radiation (1.5407 Å) while θ is the peak position. Miller indices (002) and (004) were taken for the calculation of crystallites size in the c crystallographic direction (z-axis) [48]. Bragg peaks and crystallographic planes were determined using Mercury CSD 3.10.1 software (CCDC, Cambridge, UK) from the hydroxyapatite references (No. 2300273, Crystallography Open Database, and No. 96-901-0053, High Score Plus package software). The peak position and FWHM was calculated from the fits of the Voight function to every peak with use of the OriginPro 2016 software (OriginLab Co., Northampton, MA, USA).

### Mineral content

The mineral composition of teeth was assessed using ICP-OES (iCAP Series 6500, Thermo Fisher Scientific, Waltham, MA, USA). 24 teeth crowns, previously used for chroma measurements, AFM imaging and micro hardness measurements with their corresponding roots, which were formerly cut, were examined. The teeth crowns and roots were examined separately. Prior to the analysis of mineral elements, mineralization of the samples (0.3 g for the crown and 0.3 g for the root) was conducted in a Microwave Digestion System (Berghof Speedwave, Eningen, Germany) to dissolve Cd in the presence of organic molecules. Optics, temperature and pressure were monitored for each sample during acid digestion in teflon vials (type DAP 100). The tooth sample was digested with 8 ml HNO_3_ (65% v/v) and 2 ml HCl (37% v/v). The mineralisation process followed a scheme: 10 min at the temperature increasing from room temperature to 140°C, 10 min at 140°C, 15 min at the temperature increasing from 145°C to 195°C, 10 min at 195°C and cooling down to the room temperature. The pressure has not exceeded 20 bars during the mineralisation. After the mineralisation, the clear solution, cooled to the room temperature, was transferred to 50 ml graduated flasks and filled with deionized water (18,2 MΩ, PureLab Classic, ELGA Labwater, High Wycombe, UK) to the indicator.

The operating conditions of the ICP OES were as follows: RF generator power: 1150 W, RF generator frequency: 27.12 MHz, coolant gas flow rate: 16 L/min, carrier gas flow rate: 0.65 L/min, auxiliary gas flow rate: 0.4 L/min, maximum integration time: 1 s, pump rate: 50 rpm, viewing configuration—axial, replicate—3, flush time: 20 s. TraceCERT multi-element stock solution (Sigma-Aldrich, St. Louis, MO, USA) was used to prepare the standards: Ca, Cd, Cu, Mg, Fe, P, and Zn in 10% HNO_3_−10 mg/L. The results obtained on the apparatus were calculated from μg/ml to mg/kg considering the weight of the sample for mineralization and dilution. The limits of detection and the recovery rates for the examined elements are presented in Table 1.

**Table 1:** Limits of detection and recovery efficiency for the examined elements.

Element	Coefficient of correlation r	Limit of detection LOD [μg/L]	Recovery [%]
Ca	0.9985	0.002	105
Cd	0.9999	0.001	97
Cu	0.9999	0.002	103
Fe	0.9998	0.021	96
Mg	0.9953	0.005	104
P	0.9997	0.120	103
Zn	0.9998	0.010	102

### Data analysis

Statistical analysis was performed using Statistica13.1 (TIBCO Software Inc., Palo Alto, CA, USA) and Origin2016 (OriginLab, Northampton, MA, USA) applications. After removal of outliers, the resulting dataset was checked for normality distribution by Shapiro-Wilk test and correlations among variables were determined. The analysis of variance (ANOVA) was used to detect significant factors in a multi-factor (two or three) model (Eq 8).

$$x_{ijk}=\mu +\alpha_{i}+\beta_{j}+\gamma_{k}+{\left(\alpha \beta \right)}_{ij}+{\left(\alpha \gamma \right)}_{ik}+{\left(\beta \gamma \right)}_{jk}+{\left(\alpha \beta \gamma \right)}_{ijk}+\varepsilon_{ijkl},$$ (8)

where: x_ijk_—an observation (tooth parameter), i–the first factor (group: control and cadmium), j–the second factor (sex: male or female), k–the third factor (sample location: crown or root), l–measurement number, μ –constant, α_i_−the main effect of the first factor i^th^ level, β_j_−the main effect of the second factor j^th^ level, γ_k_−the main effect of the third factor k^th^ level, (αβ)_ij_−the effect of interactions between the first and the second factor, (αγ)_ik_−the effect of interactions between the first and the third factor, (βγ)_jk_−the effect of interactions between the second and the third factor, (αβγ)_ijk_−the effect of interactions between the first, the second and the third factor, ε_ijkl_−random error. A two factor model was used to determine the effect of two main factors, that is cadmium and sex on the physical and mechanical parameters of the examined teeth (colour, mass, thickness, pigmented enamel length, roughness, hardness and fracture force), while three-way ANOVA was applied in the evaluation of the mineral content dependence on cadmium (the first factor), sex (the second factor) and sample location (the third factor). To determine which groups differ from each other post-hoc analysis (Tukey test) was applied and p-values less than 0.05 were considered as statistically significant.

## Results

### Chroma measurements

**Fig 3:**
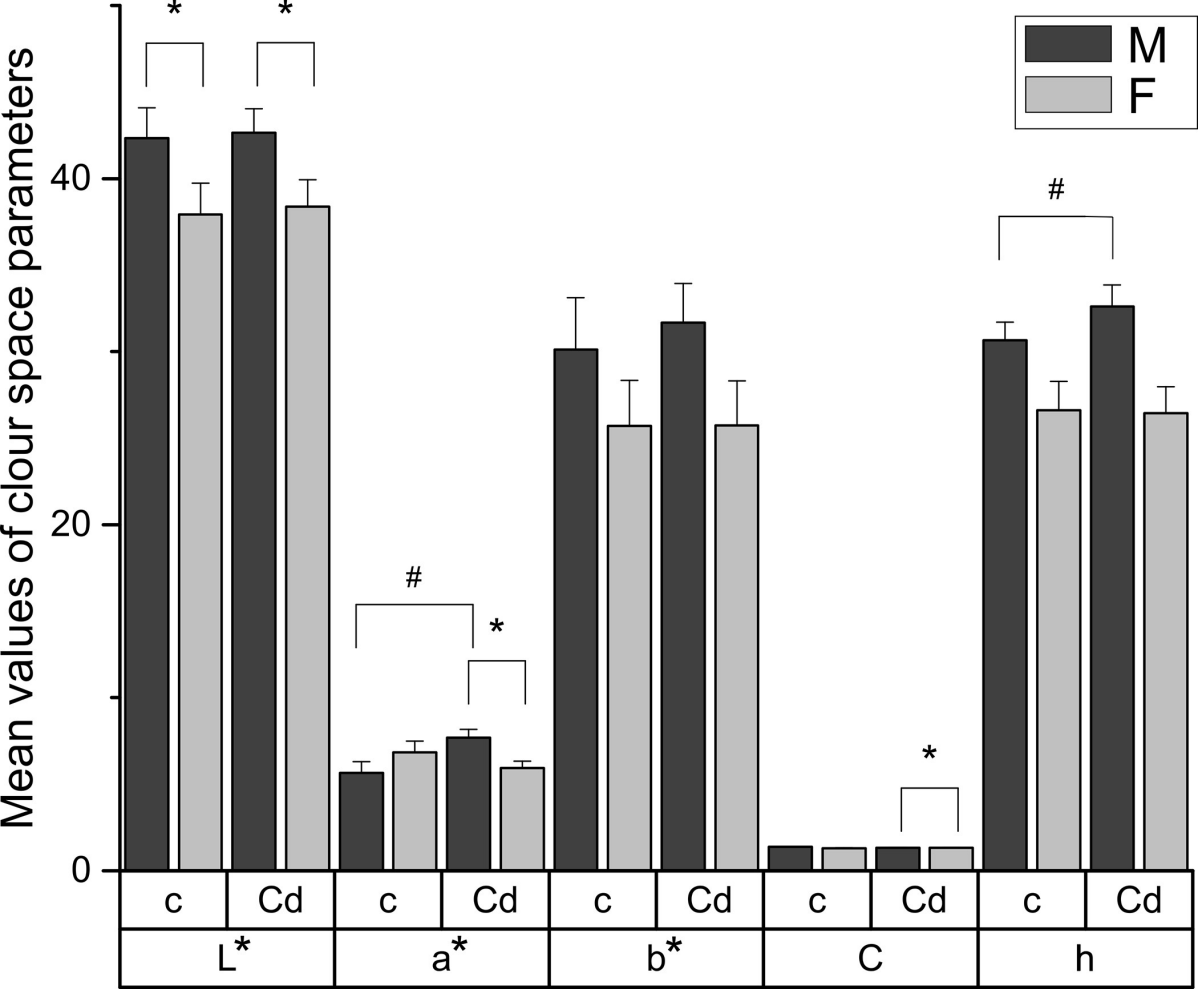
Mean values of CIE Lab colour space parameters with their standard deviations. Statistically significant differences are marked with #—significant difference between the control and the cadmium group according to the sex (p < 0.05), *–significant difference between sexes in the control and the cadmium group (p < 0.05). c–control group, Cd–cadmium group M–male, F–female L*–lightness, a*–green/red coordinate, b*–yellow/blue coordinate, C–chroma, h–hue Pigmented enamel colour parameters with a corresponding descriptive statistics–see S1 Table.

Enamel chroma measurements (Fig 3) revealed that L* and b* did not differ significantly between the control and the cadmium exposed group, however, a notable increase of a* (p = 0.001) and h (p = 0.006) values for the male cadmium group comparing to control were noticed. Sex-related changes were observed in the case of L*, which was significantly higher for males than females (p = 0.016 and p = 0.020 for the control and the cadmium group, respectively).

Males and females from the cadmium group were also notably different in terms of C (p = 0.046) and a* (p = 0.005) parameters. For b*, a slight shift into yellow chroma was registered, nonetheless the change was not statistically significant. Cadmium intoxication did not cause perceivable colour modifications of teeth between male and female sex for the control and the cadmium group, and neither did it do so for the females from the cadmium group in relation to the control (Table 2). The recognizable difference was observed only for male cadmium group comparing to the corresponding male control (NBS = 2.36).

**Table 2:** ΔE (bottom left) and NBS values (upper right) calculated for CIE Lab colour space. Differences between the control and the cadmium groups are presented on the grey background.

		Control		Cadmium	
			M	F	M	F
Control	M	-	6.35	2.36	5.42
	F	5.84	-	7.05	0.92
Cadmium	M	2.57	7.66	-	7.50
	F	5.90	1.00	6.90	-

M–male, F–female

### Morphological and mechanical parameters of teeth

The effect of sex on tooth mass, thickness and pigmented enamel length was observed. Tooth mass, thickness and pigmented enamel length were significantly greater in males, as shown in Table 3. The tooth mass and pigmented enamel length were also dependent on the treatment and were lower in the Cd-exposed groups (Table 3).

**Table 3:** Mean values of mass, thickness, length, roughness, hardness and fracture force calculated for the enamel surface for the control and the cadmium group with corresponding post-hoc analysis results.

	m [mg]	T [mm]	L [mm]	S_a_ [nm]	S_q_ [nm]	H [GPa]	F [N]
**Group**							
c	0.111	2.77	14.95	98.83	122.11	3.75	121.58
Cd	0.107	2.72	14.41	127.98	145.92	4.09	134.96
**Sex**							
M	0.124	2.86	14.07	128.62	141.74	3.94	158.12
F	0.097	2.65	14.33	99.46	121.87	3.88	105.07
**Exposure**							
M c	0.129^b^	2.89	15.77	99.75^a^	124.35^a^	3.59	139.45^b^
F c	0.098^a^	2.68	14.37	97.76^a^	119.54^a^	3.51	109.08^a^
M Cd	0.120^b^	2.84	14.52	157.45^b^	171.60^b^	4.07	172.64^c^
F Cd	0.097^a^	2.62	14.29	101.15^a^	124.20^a^	4.10	101.05^a^
**Main effects and interactions**							
group	0.056	0.199	0.030	0.111	0.087	0.027	0.131
sex	<0.001	<0.001	0.009	0.089	0.088	0.899	<0.001
group × sex	0.040	0.988	0.054	0.034	0.043	0.829	0.002

c–control, Cd–cadmium group

M–male, F–female

a, b, c–groups which do not differ at p < 0.05

m–mass, T–thickness, L–pigmented enamel length, S_a_−average roughness, S_q_−root mean square roughness, H–hardness, F–fracture force.

Mean values with a corresponding descriptive statistics–see S2 Table.

There was an interaction of both main factors on the tooth fracture force. While generally males were characterized by stronger incisors than females, for males, the tooth strength increased in the Cd-exposed group, while there was no effect on the tooth fracture force for the females (Table 3). It was also revealed that mass and thickness influenced the fracture force with correlation at the level of 0.67 and 0.44, respectively.

**Fig 4:**
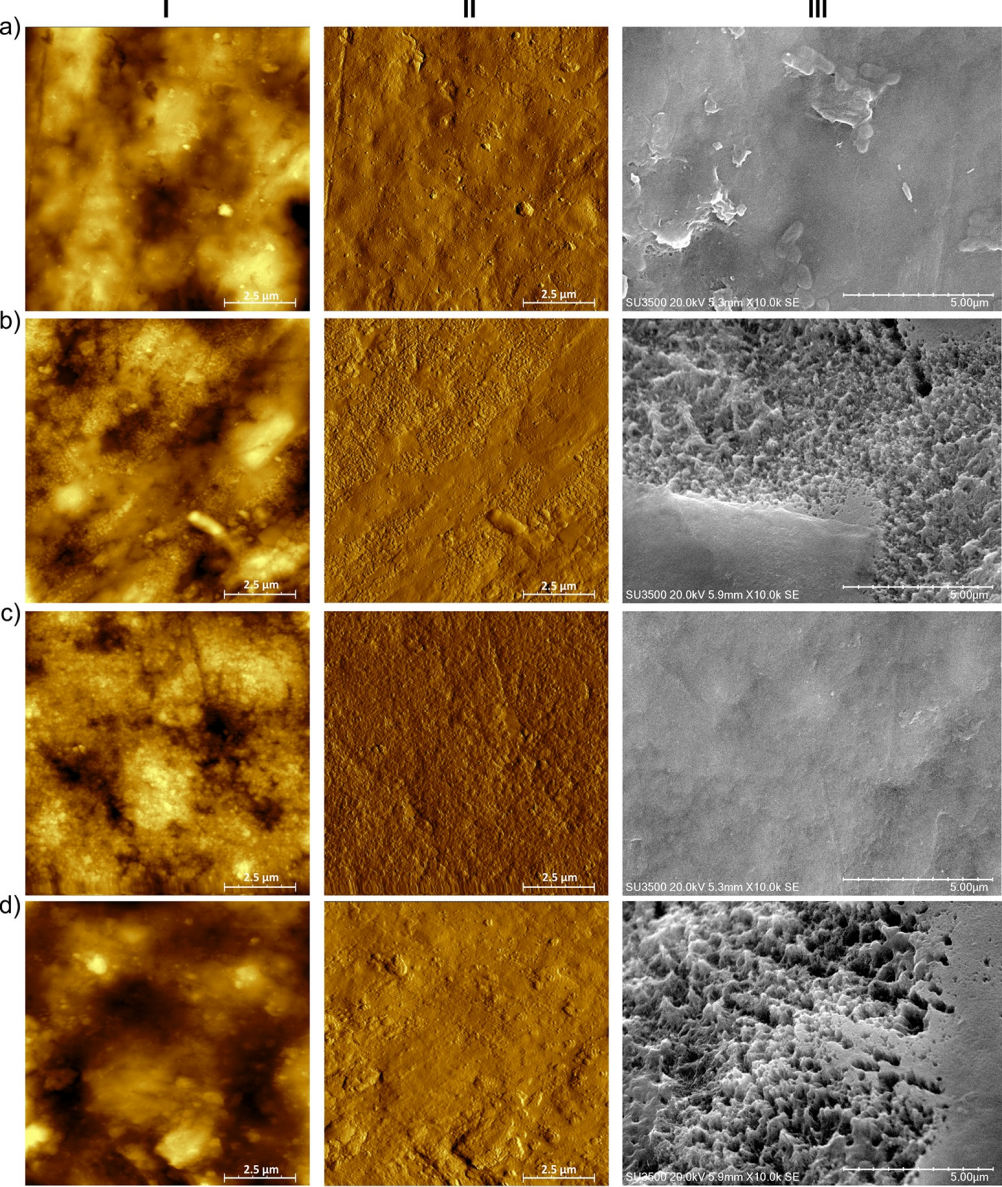
AFM scans of enamel surface in height (I) and magnitude (II) domains and corresponding SEM images (III) for female control (a), female cadmium group (b), male control (c) and male cadmium group (d).

**Fig 5:**
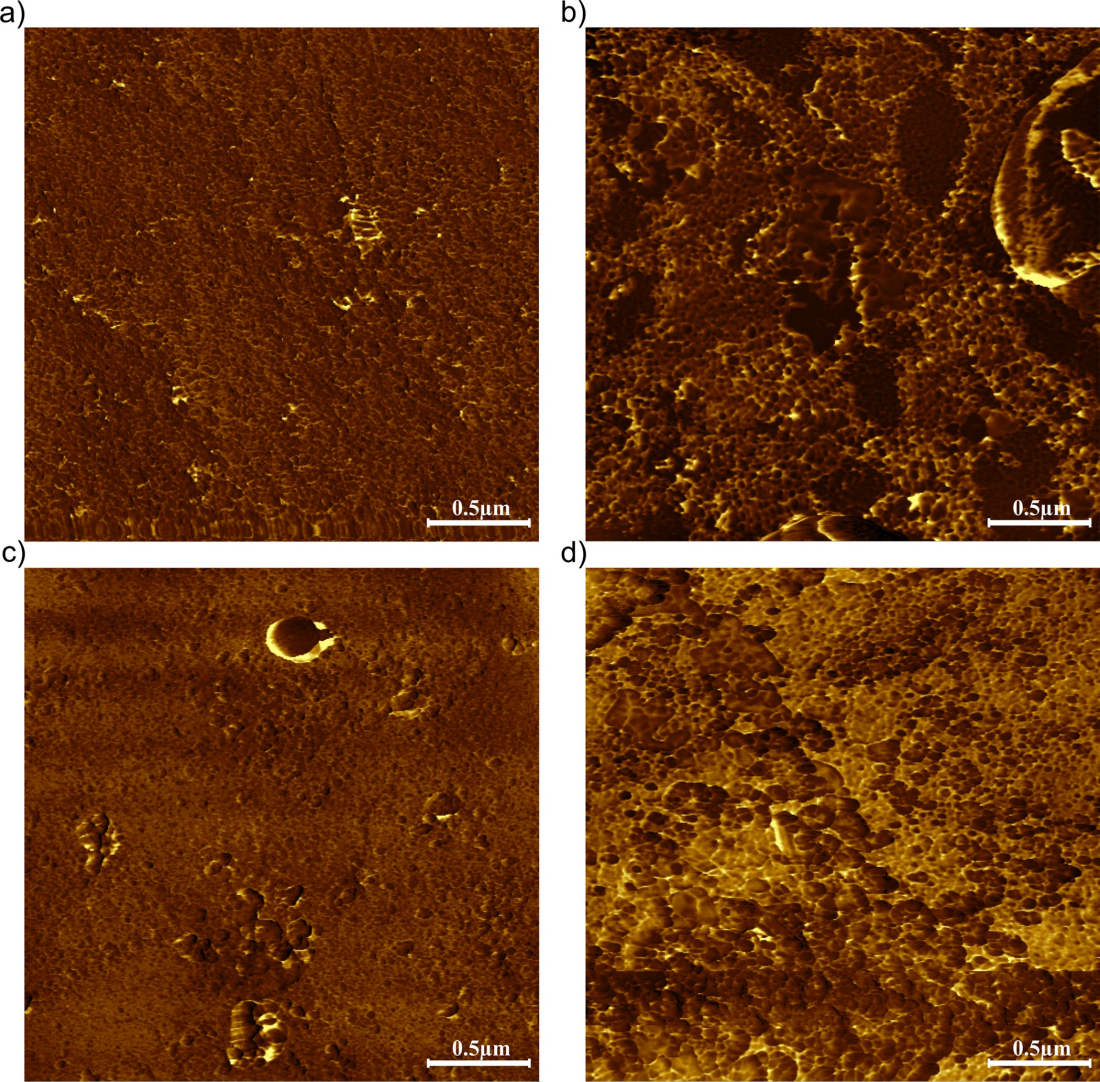
AFM phase images of enamel surface for the male control (a) and the cadmium exposed group (b), and for the female control (c) and the cadmium group (d).

There was also an interaction of the main factors on the roughness parameters (average roughness and root mean square roughness). Both roughness parameters were significantly higher for the males from the Cd group, which indicates that enamel surface in that group was more irregular comparing to the male control and both female groups (Table 3, Fig 4C and 4D). AFM phase images revealed more porous, sponge-like enamel surface in both Cd-exposed groups (Fig 5B and 5D, for the Cd male and Cd female group, respectively) next to control (Fig 5A and 5C, for the control male and control female group, respectively), however the changes in the tooth’s outer structure in Cd-exposed specimens were more evident for males.

**Fig 6:**
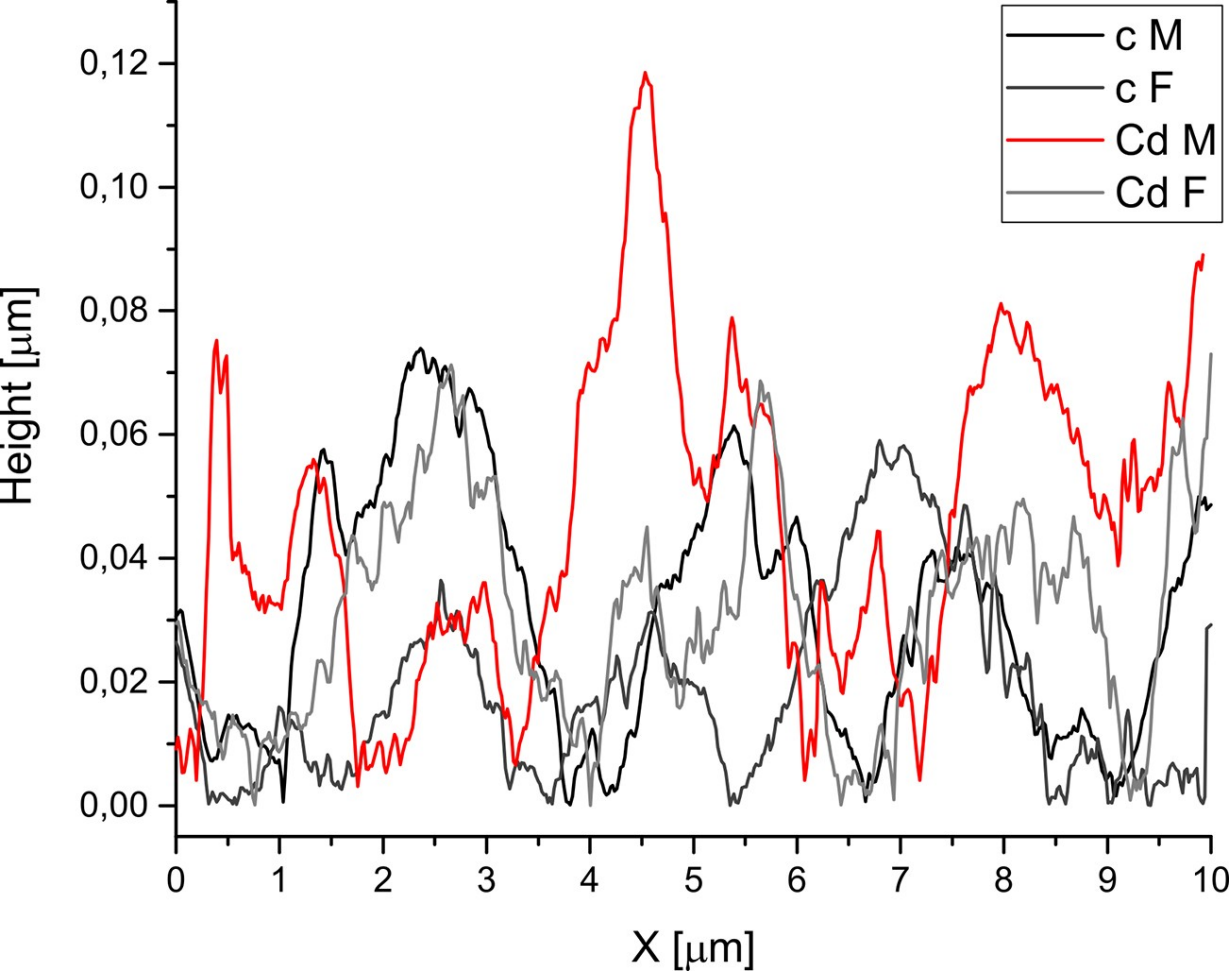
Average height profiles of tooth’s enamel surface for the control and cadmium groups according to the sex. c–control, Cd–cadmium group, M–male, F–female.

The analysis of AFM surface profile images (Fig 6) proved that average height differences on the distance of 10 μm (scan dimension) between the male control and the male cadmium group were greater than between the female control and the female Cd group (0.033 μm and 0.019 μm, respectively).

Micro hardness (Table 3) was only dependent on cadmium exposure, irrespective of the sex, and was significantly higher for the cadmium groups.

### The structure of HA crystals

**Fig 7:**
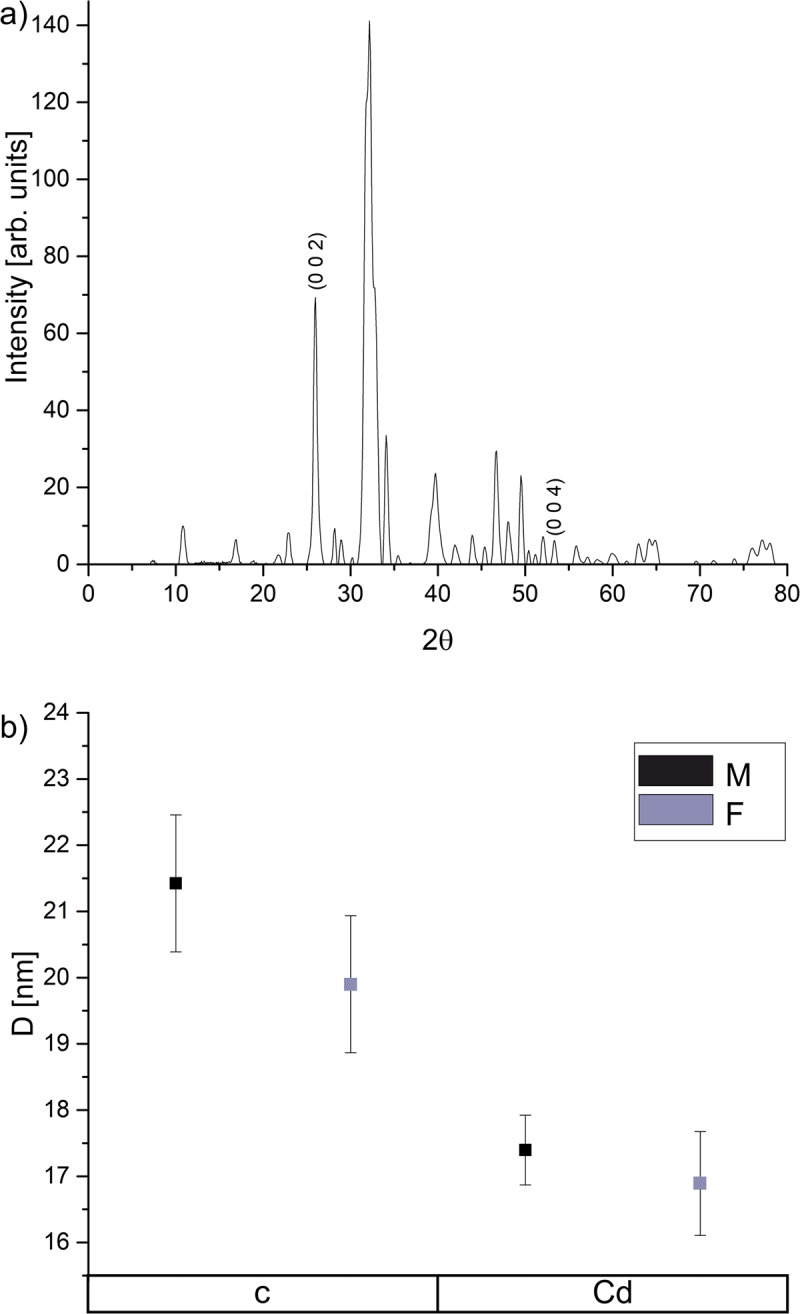
X-ray diffraction pattern of enamel HA crystals with marked reflection peaks measured from a single tooth (a) and mean crystal size of HA crystallites in c-axis with corresponding standard deviations (b) in examined groups and according to sex. c–control, Cd–cadmium exposed group, M–male, F–female.

Reflection peaks at 2θ = 25.886 deg (002) and 2θ = 53.224 deg (004) from XRD patterns (Fig 7A) were used to determine the HA crystallites length. HA crystallites in the groups exposed to cadmium rendered significantly shorter in the c crystallographic direction of about 4.03 nm for the male sex and 3.01 nm for the female individuals when compared to the corresponding controls (Fig 7B).

### Mineral content analysis

ICP-OES analysis (Table 4) revealed that cadmium intoxication caused a significant increase in the Cd content for the tooth’s crown in both sexes while for the roots its level remains unchanged comparing to the controls. However, for the Cd-exposed groups, Cd level in the tooth crown was over two times higher for the male group comparing to the female. The Cd/Ca ratio remained at the same level in the control group, irrespective of the sex, and amounted, on average, to 2.5·10^−7^, lower values were observed in the control groups for the root (10^−7^). Cadmium exposure caused a significant increase of the Cd/Ca ratio in the crowns of teeth both for male and female, however the rise of the ratio registered for males was over twice as high as for females.

**Table 4:** Mineral content in analysed teeth divided according to group, sex and sample location with the corresponding results of post-hoc analysis.

Treatment	Ca [mg/kg]	Cd [mg/kg]	Cu [mg/kg]	Fe [mg/kg]	Mg [mg/kg]	P [mg/kg]	Zn [mg/kg]	Ca/P	Cd/Ca (·10^−7^)
**Group**									
c	233240	0.047	0.705	185.40	10720	123252	110.09	1.899	1.986
Cd	228600	1.253	0.927	206.00	10113	122108	124.52	1.876	51.838
**Sex**									
M	233336	1.001	0.605	175.25	11654	122822	109.34	1.901	39.286
F	228589	0.373	1.023	215.55	9211	122519	125.56	1.864	15.962
**Sample location**									
crown	2419123	1.997	0.755	303.19	12034	127706	120.68	1.894	82.236
root	225829	0.052	0.848	146.80	9669	120366	115.98	1.876	2.326
**Exposition**									
M c crown	248150	0.067^a^	0.707	279.40	12630^a^^,^^b^	130050^b^	107.78	1.909^a^^,^^b^	2.716^a^
M c root	229933	0.022^a^	0.431	125.04	11380^a^^,^^b^	119689^a^	98.02	1.921^b^	0,978^a^
F c crown	238950	0.126^a^	0.807	332.75	11490^a^^,b^	129225^b^	126.75	1.849^a^^,^^b^	5.258^a^
F c root	226650	0.025^a^	0.962	132.48	8638^c^	120875^a^	116.50	1.875^a^^,^^b^	1.086^a^
M Cd crown	244325	5.780^c^	0.604	287.65	12783^b^	126950^b^	125.93	1.924^a^^,^^b^	235.453^c^
M Cd root	224538	0.058^a^	0.749	123.23	10924^a^	120675^a^	113.53	1.861^a^	2.585^a^
F Cd crown	236225	2.013^b^	0.901	312.98	11235^a^^,^^b^	124600^a^^,^^b^	122.25	1.896^a^^,^^b^	85.516^b^
F Cd root	222510	0.094^a^	1.210	196.69	7949^c^	120320^a^	133.67	1.849^a^	2.322^a^
**Main effects and interactions**									
group	0.038	<0.001	0.093	0.292	0.205	0.029	0.058	0.587	<0.001
sex	0.009	<0.001	<0.001	0.002	<0.001	0.470	0.017	0.002	<0.001
sample location	<0.001	<0.001	0.309	<0.001	<0.001	<0.001	0.338	0.109	<0.001
group × sex	0.777	<0.001	0.702	0.430	0.513	0.346	0.338	0.145	<0.001
group × sample location	0.719	<0.001	0.082	0.128	0.288	0.015	0.385	0.001	<0.001
sex × sample location	0.154	<0.001	0.073	0.963	0.003	0.220	0.288	0.505	<0.001
group × sex × sample location	0.985	<0.001	0.413	0.055	0.857	0.996	0.268	0.943	<0.001

c–control, Cd–cadmium group

M–male, F–female

a, b, c, d–groups which do not differ at p < 0.05

Mean values of mineral content according to group, sex and tooth’s fragment with corresponding descriptive statistics–see S3 Table.

In the case of Ca and P significant differences between root and crown, with a significantly higher content in crown, were identified. Ca and P contents were dependent on cadmium exposition. In Cd-exposed group significantly lower values of Ca and P were observed. The Ca/P ratio was stable among all the examined groups, however the difference between Cd male group (Ca/P = 1.861) and the corresponding male control for the root fragment (Ca/P = 1.921) was observed. The Cu, Zn and Mg levels remained stable, irrespective of Cd exposition. Cd exposure has not caused notable differences in the Fe content either.

## Discussion

Cadmium is a toxic metal with a biological half-life of 10–30 years. It is not degradable, and does not decay and accumulates in the organisms, where it gives rise to a number of adverse health effects, such as nephrotoxicity and bone damage [49, 50]. Long-term exposure to Cd causes toxic effects, especially in the liver and kidneys, and can change the structure of intestinal mucosa [51]. In rats exposed to Cd reduction of bone geometrical parameters, mechanical endurance, densitometry, trabecular bone histomorphometry, as well alteration of the structure of articular and growth plate cartilages were observed [10, 13, 52]. Exposure to cadmium also impacts teeth enamel. Its action covers, among others, inhibition of proteinolysis process [25] and triggering crystal defects such as perforations in developing tooth enamel [53]. Cadmium also decreases alkaline phosphatase activity and mineralization process [14] and causes perturbation of the vitamin D metabolic pathway, which leads to a higher caries prevalence [20]. Such actions may cause variations in the arrangement and size of hydroxyapatite crystals and thereby alter enamel colour, structure and mechanical features [23, 26, 54, 55].

The presented study showed that cadmium exposure gave rise to a range of structural and morphological changes in the pigmented enamel of Wistar rats’ incisors. The surface of the pigmented enamel was marked by shift in the colour, reduction in the length, increase in hardness and greater irregularity (Table 3, Figs 3 and 4). Oral intake of cadmium by adult rats also caused a significant rise in Cd levels in the teeth’s crowns (Table 4) and decrease in crystallites size in the c axis. The most apparent changes were observed for males.

Mechanical properties of teeth, such as micro hardness or elastic modulus are tightly connected with the enamel structure and the content of calcium and organic matter in the tissues of teeth [56, 57]. In their work, Eimar et al. [55] shown that enamel hardness was associated with the size of apatite crystals along the c-axis, which might be influenced, among others, by the introduction of trace elements. The presence of some of them, such as Co or Ti [58], increases enamel hardness [23], while for others, such as Pb, no action on its mechanical properties was observed [28]. However, the influence of Cd intoxication on enamel hardness has not been reported yet, the presented results show similar effect as observed for Co and Ti. An increase in hardness with connection of alteration of HA crystallite size in the c-axis may lead to the conclusion, that crystal growth was probably disturbed, which contributed to enamel structural alterations, as evidenced by a greater surface roughness (Table 3). The mechanism responsible for this action is probably multifactorial in origin and may be explained by incorporation of Ca^2+^ ions into the enamel’s structure with simultaneous Cd interaction with enamel matrix proteins and/or proteinases. Even though Ca and Cd differ in terms of chemical properties, both are divalent cations and have the same overall charge [27] and similar radii (Ca^2+^ = 0.99 Å, Cd^2+^ = 0.97 Å) [26]. As a consequence, Cd^2+^ is recognised as a steric mimic for calcium and can easily replace Ca^2+^ in HA, causing general shortening of the interatomic distances both in the a- and c-axis, as shown in numerous *in vitro* research [24, 26, 53]. Additionally, in biological systems Cd^2+^ is able to interact with proteins and proteinases, which are responsible for a range of structural processes. For example, Gerlach et al. [25] observed *in vivo* that the total proteolytic activity of enamel matrix decreased significantly when incubated with Cd solution. Proteinases (metalloproteinase-20, MMP-20 and kallikrein-related peptidase-4, KLK4) play a key role during dental enamel formation by processing and degradation of enamel proteins [4, 59] and any dysfunctions in MMP-20 and KLK4 result in creating porous enamel, containing residual proteins [60]. It was also proved that Cd^2+^ influences the crystal nucleation process by lowering the activity of carbonic anhydrase, which leads to the insufficient supply of carbonate ions, which are responsible for crystal growth [53]. In contrast, in the other study it was shown that enamel has little affinity to cadmium and thus only takes up a very slight amount of it [61]. It should be also underlined that pigmented enamel is characterised by the presence of a ferrihydrite (Fh) and amorphous iron-calcium phosphate. Ferrihydrite pockets are present as a completely separated intergranular phases of Fe-enriched enamel [62, 63]. As cadmium interferes with the iron and phosphatases metabolism [21, 64] and higher amounts of Cd were detected in the crowns, it can be also suspected that Cd^2+^ ions might be positioned in Fh pockets.

Our research has shown an increase of the fracture force for incisors of males from the cadmium group. The rise observed in our study (Table 3) contradicts the presented outcomes observed for bone tissue, for which a decrease in the mechanical and biomechanical properties of bones was registered after cadmium exposure. For example, rats co-exposed to Cd and Pb for 12 weeks were characterized with a reduced values of mechanical endurance (ultimate strength and maximum elastic force) and geometric parameters (length and cross section area) [13]. Female rats exposed to cadmium for up to 24 months in low and moderate doses (1 and 5 mg Cd/kg) were characterized with a decrease in yield strength in the case of femoral neck and a decline in yield strength, yield stress, stiffness and fracture strength of femoral diaphysis [65]. Tomaszewska et.al. [14] also showed that bones of rats exposed to Cd and Pb diet (7 mg Cd/kg_bw_ and 50 mg Pb/kg_bw_) for 12 weeks were characterized with reduced Ca content, lower mechanical endurance and altered histomorphometry of trabecular bone. However, changes observed in the presented study are probably not connected to cadmium action but mainly to the sex effect, as significant differences between female and male specimens were registered. Moreover, tooth fracture force was positively correlated with the tooth mass and thickness, which were also sex-dependent.

Increased content of trace elements in enamel causes variations not only in the mechanical properties but also in the optical ones. Incorporation of Fe^2+^ ions leads to black pigmentation of teeth, after Mn^2+^ exposure enamel becomes brown or black whereas the presence of Cd^2+^ may cause yellow staining [22, 23]. In the presented research the pigmented enamel from the male cadmium group was characterized by a slight increase in a* and b* values (into red and yellow, respectively, Fig 2). It caused perceivable changes in colour in comparison to the control (ΔE = 2.57, Table 2). When rats were injected with Cd intravenously with doses of 1.0 and 2.0 mg/kg, 5 days/week, discolouration of the incisors was observed and for rats exposed to 2.0 mg/kg_bw_ their teeth colour shifted from yellowish into ivory [21]. Moreover, histopathologic examination of the incisors demonstrated decreased iron-containing pigment in ameloblasts and destruction of the enamel organ. The authors suggest that the observed changes were caused by the Fe^2+^ decrease, as lower Fe content was detected in the cadmium group and that the pigment present in the cytoplasm of ameloblasts was directly displaced by Cd ions. In our work the Fe content was not dependent on Cd treatment (Table 4) and such significant alterations in the enamel colour were not observed (Fig 2, Table 2), however, a slight shift of the hue from orange into yellow (from 30.65 to 32.60) was noted. Alterations in the colour might be also related to increased surface roughness as roughness plays an important role on the light scattering pattern [66]. Along with an increase in roughness, higher scattering is observed, whereby lower wavelengths are scattered to a greater degree.

The results obtained in the presented study show a great disproportion in Cd levels and enamel structural alterations between male and female rats. As shown in numerous studies, females are usually more sensitive to Cd-induced alterations, especially in a the case of skeletal and nervous systems [67], development of reproductive disorders and risk of cancer [68]. Thus, we have assumed that some differences might occur as discrepancies in Cd toxicity and Cd tissue distribution among the sexes had been reported [69, 70]. However, the achieved effect was opposite to what we expected. Our findings show that the response to Cd exposure was greater for males and the cadmium-induced changes of the enamel surface were more visible. Moreover, in our research the content of Cd in Cd-exposed groups was notably higher in males whereas no or minor differences in Cd content in hard tissues of teeth and bones between males and females are described in other works. Roczaniak et al. [71] analysed the content of cadmium, nickel, copper and zinc in tibia, femur and meniscus in men and women who underwent a knee replacement surgery. No statistically significant differences in the content of the examined elements was registered, however, in the case of females, lower Cd values were noted in comparison to males. In animal studies, it was shown, that young male Wistar rats are less vulnerable to the effects of Cd compared to the females, for which osteopenia and high bone turnover with increased resorption were ascertained [18, 72]. Alomary et. al [73] measured the concentrations of lead (Pb), cadmium (Cd), copper (Cu), iron (Fe), and zinc (Zn) in deciduous teeth from children living in Jordan, and no sex-dependent differences for Cd concentration were ascertained. No effect of Cd levels according to sex was also observed by Kumagai et al. [74], where the content of elements in the dentin was measured according to sex and age.

Concluding, Cd intake exerted a number of structural and chemical effects on the properties and formation of hydroxyapatite which translated into changes in the enamel morphology and mechanical features. The range of structural alterations caused by cadmium and its multiway and multi-levelled action on the biological systems requires more detailed studies and analysis, including high-resolution analytical techniques (EDX, APT). As it is not apparent from the present study that cadmium acts on the organic or inorganic part of the teeth tissue or simultaneously on the both phases, additional experiments are needed to ambiguously assess the nature of cadmium impact. The results suggest, that cadmium presence in the tissue of the teeth disturbed hydroxyapatite crystals growth and influenced their size. The presence of cadmium ions altered colour, structure and mechanical features of the pigmented enamel. As the general pattern of cadmium distribution is approximately the same in laboratory animals and humans, our results, to some extent, could be related to the man concerning indicators and target organs for trace elements.

## Supporting information

S1 Table Pigmented enamel colour parameters with corresponding descriptive statistics. c–control group, Cd–cadmium group M–male, F–female, L*–lightness, a*–green/red coordinate, b*–yellow/blue coordinate, C–chroma, h–hue.
(DOCX)

S2 Table Mean values of mass, thickness, length, roughness, hardness and fracture force calculated for the enamel surface for the control and cadmium group according to sex with corresponding descriptive statistics. c–control, Cd–cadmium group, M–male, F–female, m–mass, T–thickness, L–enamel length, S_a_−average roughness, S_q_−root mean square roughness, H–hardness, F–fracture force.
(DOCX)

S3 Table Mean values of mineral content in analysed teeth divided according to group, sex and tooth’s fragment with corresponding descriptive statistics. c–control, Cd–cadmium group, M–male, F–female.
(DOCX)
