# Supplementary material for: The effect of cadmium exposition on the structure and mechanical properties of rat incisors
Source: PLoS One. 2019 Apr 12;14(4):e0215370. doi: 10.1371/journal.pone.0215370 (PMC6461291; doi:10.1371/journal.pone.0215370)
Supplement: S1 Table — c–control group, Cd–cadmium group M–male, F–female, L*–lightness, a*–green/red coordinate, b*–yellow/blue coordinate, C–chroma, h–hue. (DOCX) [file pone.0215370.s001.docx]

S1 Table. Pigmented enamel colour parameters with corresponding descriptive statistics.

| **Colour parameter** | **Group** | **Sex** | **N teeth** | **N data points** | **Mean** | **Standard deviation** | **Minimum** | **Median** | **Maximum** |
| --- | --- | --- | --- | --- | --- | --- | --- | --- | --- |
| L* | c | F | 6 | 17 | 42.32 | 1.77 | 33.75 | 42.35 | 47.98 |
|  |  | M | 6 | 16 | 37.91 | 1.81 | 29.34 | 39.10 | 42.66 |
|  | Cd | F | 6 | 18 | 42.65 | 1.39 | 39.60 | 41.98 | 46.76 |
|  |  | M | 6 | 14 | 38.38 | 1.57 | 29.18 | 38.88 | 41.59 |
| a* | c | F | 6 | 14 | 5.65 | 0.67 | 5.80 | 6.55 | 7.90 |
|  |  | M | 6 | 16 | 6.84 | 0.66 | 5.94 | 7.94 | 9.45 |
|  | Cd | F | 6 | 15 | 7.68 | 0.51 | 6.47 | 7.36 | 9.04 |
|  |  | M | 6 | 14 | 5.95 | 0.39 | 5.43 | 5.90 | 7.43 |
| b* | c | F | 6 | 14 | 30.12 | 2.99 | 28.28 | 32.26 | 38.98 |
|  |  | M | 6 | 18 | 25.71 | 2.63 | 26.33 | 29.32 | 34.20 |
|  | Cd | F | 6 | 17 | 31.67 | 2.26 | 22.97 | 31.32 | 36.76 |
|  |  | M | 6 | 16 | 25.75 | 2.55 | 20.49 | 27.38 | 31.27 |
| C | c | F | 6 | 14 | 1.39 | 0.01 | 1.33 | 1.39 | 1.42 |
|  |  | M | 6 | 18 | 1.31 | 0.01 | 1.28 | 1.31 | 1.36 |
|  | Cd | F | 6 | 17 | 1.33 | 0.02 | 1.29 | 1.33 | 1.38 |
|  |  | M | 6 | 16 | 1.34 | 0.03 | 1.17 | 1.34 | 1.37 |
| h | c | F | 6 | 14 | 30.65 | 1.04 | 28.84 | 32.85 | 39.64 |
|  |  | M | 6 | 18 | 26.60 | 1.69 | 27.06 | 30.42 | 35.48 |
|  | Cd | F | 6 | 17 | 32.59 | 1.26 | 23.86 | 32.48 | 37.55 |
|  |  | M | 6 | 18 | 26.44 | 1.53 | 21.38 | 27.95 | 32.13 |

c – control group, Cd – cadmium group

M – male, F – female

L* – lightness, a* – green/red coordinate, b* – yellow/blue coordinate, C – chroma, h – hue
