# Supplementary material for: The effect of cadmium exposition on the structure and mechanical properties of rat incisors
Source: PLoS One. 2019 Apr 12;14(4):e0215370. doi: 10.1371/journal.pone.0215370 (PMC6461291; doi:10.1371/journal.pone.0215370)
Supplement: S2 Table — c–control, Cd–cadmium group, M–male, F–female, m–mass, T–thickness, L–enamel length, Sa−average roughness, Sq−root mean square roughness, H–hardness, F–fracture force. (DOCX) [file pone.0215370.s002.docx]

S2 Table. Mean values of mass, thickness, length, roughness, hardness and fracture force calculated for the enamel surface for the control and cadmium group according to sex with corresponding descriptive statistics

| **Physical parameter** | **Group** | **Sex** | **N teeth** | **N data points** | **Mean** | **Standard deviation** | **Minimum** | **Median** | **Maximum** |
| --- | --- | --- | --- | --- | --- | --- | --- | --- | --- |
| m [mg] | c | F | 12 | 12 | 0.098 | 0.003 | 0.092 | 0.099 | 0.102 |
|  |  | M | 12 | 12 | 0.129 | 0.005 | 0.121 | 0.131 | 0.134 |
|  | Cd | F | 12 | 12 | 0.097 | 0.005 | 0.091 | 0.094 | 0.107 |
|  |  | M | 12 | 12 | 0.120 | 0.007 | 0.110 | 0.117 | 0.134 |
| T [mm] | c | F | 12 | 12 | 2.68 | 0.08 | 2.58 | 2.67 | 2.83 |
|  |  | M | 12 | 12 | 2.89 | 0.23 | 2.69 | 2.85 | 3.39 |
|  | Cd | F | 12 | 12 | 2.62 | 0.11 | 2.48 | 2.57 | 2.79 |
|  |  | M | 12 | 12 | 2.84 | 0.08 | 2.72 | 2.85 | 2.98 |
| L [mm] | c | F | 12 | 12 | 14.37 | 0.86 | 12.79 | 14.28 | 15.54 |
|  |  | M | 12 | 12 | 15.77 | 0.73 | 14.99 | 15.94 | 16.68 |
|  | Cd | F | 12 | 12 | 14.29 | 1.18 | 12.70 | 14.20 | 16.65 |
|  |  | M | 12 | 12 | 14.52 | 0.46 | 13.83 | 14.70 | 15.02 |
| S_a_ [µm] | c | F | 6 | 30 | 97.78 | 28.00 | 64.89 | 75.60 | 193.4 |
|  |  | M | 6 | 28 | 99.75 | 28.74 | 63.05 | 101.83 | 147.91 |
|  | Cd | F | 6 | 27 | 101.15 | 18.80 | 72.26 | 100.49 | 127.05 |
|  |  | M | 6 | 30 | 157.45 | 35.41 | 127.12 | 155.28 | 188.75 |
| S_q_ [µm] | c | F | 6 | 28 | 119.54 | 17.47 | 80.08 | 92.06 | 218.31 |
|  |  | M | 6 | 30 | 124.35 | 22.06 | 84.61 | 125.13 | 175.81 |
|  | Cd | F | 6 | 30 | 124.20 | 21.82 | 87.85 | 125.57 | 149.34 |
|  |  | M | 6 | 26 | 174.93 | 38.43 | 160.31 | 220.42 | 265.71 |
| H [GPa] | c | F | 6 | 30 | 3.51 | 0.55 | 3.18 | 3.50 | 5.02 |
|  |  | M | 6 | 25 | 3.59 | 0.89 | 2.95 | 3.56 | 6.32 |
|  | Cd | F | 6 | 30 | 4.10 | 0.81 | 2.91 | 4.12 | 5.34 |
|  |  | M | 6 | 27 | 4.07 | 0.95 | 3.03 | 3.60 | 6.06 |
| F [N] | c | F | 6 | 6 | 109.08 | 8.93 | 95.59 | 107.31 | 120.46 |
|  |  | M | 6 | 6 | 139.45 | 21.21 | 105.06 | 127.98 | 163.24 |
|  | Cd | F | 6 | 6 | 101.05 | 9.04 | 89.79 | 101.09 | 118.39 |
|  |  | M | 6 | 6 | 172.64 | 22.11 | 156.64 | 176.22 | 219.35 |

c – control, Cd – cadmium group,

M – male, F – female,

m – mass, T – thickness, L – enamel length, S_a_ – average roughness, S_q_ – root mean square roughness, H – hardness, F – fracture force
