# Supplementary material for: The effect of cadmium exposition on the structure and mechanical properties of rat incisors
Source: PLoS One. 2019 Apr 12;14(4):e0215370. doi: 10.1371/journal.pone.0215370 (PMC6461291; doi:10.1371/journal.pone.0215370)
Supplement: S3 Table — c–control, Cd–cadmium group, M–male, F–female. (DOCX) [file pone.0215370.s003.docx]

S3 Table. Mean values of mineral content in analysed teeth divided according to group, sex and tooth’s fragment with corresponding descriptive statistics

| **Mineral** | **Group** | **Sex** | **Tooth part** | **N teeth** | **N data points** | **Mean** | **Standard deviation** | **Minimum** | **Median** | **Maximum** |
| --- | --- | --- | --- | --- | --- | --- | --- | --- | --- | --- |
| Ca | c | F | crown | 6 | 6 | 238950 | 4679 | 234600 | 237850 | 245500 |
|  |  |  | root | 6 | 5 | 226650 | 3365 | 213800 | 226000 | 239300 |
|  |  | M | crown | 6 | 5 | 248150 | 3005 | 244600 | 248050 | 251900 |
|  |  |  | root | 6 | 6 | 229933 | 2648 | 222300 | 229400 | 239500 |
|  | Cd | F | crown | 6 | 6 | 236225 | 3690 | 226300 | 237700 | 243200 |
|  |  |  | root | 6 | 6 | 222510 | 2585 | 211700 | 220400 | 239700 |
|  |  | M | crown | 6 | 5 | 244325 | 2788 | 234900 | 244850 | 252700 |
|  |  |  | root | 6 | 6 | 224538 | 3619 | 217000 | 224950 | 228400 |
| Cd | c | F | crown | 6 | 5 | 0.126 | 0.027 | 0.144 | 0.184 | 0.223 |
|  |  |  | root | 6 | 6 | 0.025 | 0.007 | 0.018 | 0.025 | 0.035 |
|  |  | M | crown | 6 | 6 | 0.067 | 0.019 | 0.043 | 0.068 | 0.089 |
|  |  |  | root | 6 | 6 | 0.022 | 0.009 | 0.013 | 0.022 | 0.029 |
|  | Cd | F | crown | 6 | 5 | 2.013 | 0.047 | 1.474 | 2.037 | 2.504 |
|  |  |  | root | 6 | 6 | 0.094 | 0.006 | 0.013 | 0.017 | 0.198 |
|  |  | M | crown | 6 | 6 | 5.780 | 0.791 | 3.101 | 6.268 | 7.482 |
|  |  |  | root | 6 | 5 | 0.058 | 0.005 | 0.036 | 0.055 | 0.095 |
| Cu | c | F | crown | 6 | 6 | 0.807 | 0.103 | 0.6701 | 0.805 | 1.249 |
|  |  |  | root | 6 | 6 | 0.962 | 0.092 | 0.5985 | 0.790 | 1.466 |
|  |  | M | crown | 6 | 5 | 0.707 | 0.039 | 0.5629 | 0.687 | 0.891 |
|  |  |  | root | 6 | 5 | 0.431 | 0.054 | 0.2055 | 0.373 | 0.648 |
|  | Cd | F | crown | 6 | 6 | 0.901 | 0.121 | 0.735 | 0.939 | 1.089 |
|  |  |  | root | 6 | 5 | 1.210 | 0.120 | 0.867 | 1.218 | 1.620 |
|  |  | M | crown | 6 | 5 | 0.604 | 0.079 | 0.524 | 0.603 | 0.785 |
|  |  |  | root | 6 | 6 | 0.749 | 0.075 | 0.572 | 0.726 | 0.893 |
| Fe | c | F | crown | 6 | 6 | 332.75 | 21.09 | 299.60 | 319.3 | 392.81 |
|  |  |  | root | 6 | 6 | 132.48 | 22.13 | 104.10 | 138.75 | 165.10 |
|  |  | M | crown | 6 | 6 | 279.40 | 18.67 | 254.22 | 272.2 | 319.65 |
|  |  |  | root | 6 | 6 | 125.04 | 12.54 | 94.45 | 129.8 | 155.00 |
|  | Cd | F | crown | 6 | 6 | 312.98 | 21.11 | 296.71 | 305.6 | 344.00 |
|  |  |  | root | 6 | 5 | 196.69 | 15.96 | 115.46 | 184.15 | 272.81 |
|  |  | M | crown | 6 | 6 | 287.65 | 20.04 | 238.41 | 252.75 | 306.74 |
|  |  |  | root | 6 | 6 | 123.23 | 11.29 | 94.67 | 121.8 | 153.51 |
| Mg | c | F | crown | 6 | 6 | 11490 | 292 | 11100 | 11525 | 11810 |
|  |  |  | root | 6 | 6 | 8638 | 284 | 7291 | 8663.5 | 10030 |
|  |  | M | crown | 6 | 6 | 12630 | 399 | 11190 | 12665 | 14000 |
|  |  |  | root | 6 | 6 | 11380 | 425 | 9823 | 11620 | 12700 |
|  | Cd | F | crown | 6 | 6 | 11235 | 422 | 10560 | 11285 | 11810 |
|  |  |  | root | 6 | 6 | 7949 | 300 | 6226 | 8068.5 | 8824 |
|  |  | M | crown | 6 | 6 | 12783 | 410 | 12240 | 12755 | 13380 |
|  |  |  | root | 6 | 6 | 10924 | 412 | 10220 | 10885 | 11670 |
| P | c | F | crown | 6 | 6 | 129225 | 1878 | 127300 | 128950 | 131700 |
|  |  |  | root | 6 | 6 | 120875 | 1345 | 114600 | 122050 | 125200 |
|  |  | M | crown | 6 | 6 | 130050 | 2179 | 127000 | 130550 | 132100 |
|  |  |  | root | 6 | 6 | 119689 | 2269 | 116400 | 120400 | 123000 |
|  | Cd | F | crown | 6 | 6 | 124600 | 2849 | 119600 | 125400 | 128000 |
|  |  |  | root | 6 | 6 | 120320 | 2283 | 117700 | 120500 | 123900 |
|  |  | M | crown | 6 | 6 | 126950 | 3145 | 124000 | 126850 | 130100 |
|  |  |  | root | 6 | 6 | 120675 | 2368 | 116800 | 121050 | 124800 |
| Zn | c | F | crown | 6 | 6 | 126.75 | 18.05 | 110.50 | 122.16 | 152.30 |
|  |  |  | root | 6 | 6 | 116.50 | 8.34 | 104.21 | 116.70 | 125.70 |
|  |  | M | crown | 6 | 6 | 107.78 | 5.378 | 103.39 | 106.70 | 114.42 |
|  |  |  | root | 6 | 5 | 98.02 | 11.78 | 85.71 | 93.66 | 119.96 |
|  | Cd | F | crown | 6 | 6 | 122.25 | 14.05 | 107.00 | 122.35 | 137.32 |
|  |  |  | root | 6 | 6 | 133.67 | 17.46 | 101.34 | 128.72 | 188.51 |
|  |  | M | crown | 6 | 6 | 125.93 | 14.51 | 100.25 | 116.91 | 169.71 |
|  |  |  | root | 6 | 6 | 113.53 | 11.71 | 102.63 | 107.35 | 133.52 |
| Ca/P | c | F | crown | 6 | 6 | 1.849 | 0.010 | 1.842 | 1.844 | 1.864 |
|  |  |  | root | 6 | 6 | 1.875 | 0.020 | 1.843 | 1.875 | 1.911 |
|  |  | M | crown | 6 | 6 | 1.909 | 0.044 | 1.851 | 1.913 | 1.956 |
|  |  |  | root | 6 | 6 | 1.921 | 0.046 | 1.850 | 1.912 | 2.019 |
|  | Cd | F | crown | 6 | 6 | 1.896 | 0.003 | 1.892 | 1.895 | 1.900 |
|  |  |  | root | 6 | 5 | 1.849 | 0.048 | 1.798 | 1.838 | 1.944 |
|  |  | M | crown | 6 | 6 | 1.924 | 0.027 | 1.886 | 1.931 | 1.947 |
|  |  |  | root | 6 | 6 | 1.861 | 0.030 | 1.830 | 1.853 | 1.915 |
| Cd/Ca | c | F | crown | 6 | 6 | 5.258 | 0.156 | 4.607 | 5.678 | 9.067 |
|  |  |  | root | 6 | 6 | 1.086 | 0.176 | 0.928 | 1.053 | 1.486 |
|  |  | M | crown | 6 | 6 | 2.716 | 0.577 | 1.722 | 2.761 | 3.617 |
|  |  |  | root | 6 | 6 | 0.978 | 0.158 | 0.137 | 0.929 | 1.684 |
|  | Cd | F | crown | 6 | 5 | 85.516 | 10.508 | 61.085 | 85.164 | 110.649 |
|  |  |  | root | 6 | 6 | 2.322 | 0.189 | 0.908 | 2.562 | 5.362 |
|  |  | M | crown | 6 | 6 | 235.453 | 28.636 | 182.013 | 249.992 | 309.813 |
|  |  |  | root | 6 | 6 | 2.585 | 0.156 | 0.880 | 1.612 | 4.850 |

c – control, Cd – cadmium group

M – male, F – female
